# Predicting One-Year Mortality after Discharge Using Acute Heart Failure Score (AHFS)

**DOI:** 10.3390/jcm13072018

**Published:** 2024-03-30

**Authors:** Mariarosaria Magaldi, Erika Nogue, Nicolas Molinari, Nicola De Luca, Anne-Marie Dupuy, Florence Leclercq, Jean-Luc Pasquie, Camille Roubille, Grégoire Mercier, Jean-Paul Cristol, François Roubille

**Affiliations:** 1 Department of Advanced Biomedical Sciences, University of Naples Federico II, 80138 Naples, Italy; m.magaldi@hotmail.it (M.M.);; 2 Cardiology Department, Montpellier University Hospital, Inserm U1046, CNRS UMR 9214, PhyMedExp, 34295 Montpellier, Francejl-pasquie@chu-montpellier.fr (J.-L.P.); 3 Clinical Research and Epidemiology Unit, University Hospital of Montpellier, Montpellier University, 34090 Montpellier, France; 4 Institute of Epidemiology and Public Health, INSERM, INRIA, CHU Montpellier, University of Montpellier, 34090 Montpellier, France; 5 Département de Biochimie et Hormonologie, Centre de Ressources Biologiques, CHU de Montpellier, 34295 Montpellier, France; am-dupuy@chu-montpellier.fr; 6 PhyMedExp, University of Montpellier, INSERM U1046, CNRS UMR 9214, CEDEX 5, 34090 Montpellier, France; 7 Department of Internal Medicine PhyMedExp CHU Montpellier, Montpellier University, 34090 Montpellier, France; 8 Department of Statistics, Montpellier University Hospital, CEDEX 5, 34090 Montpellier, France; g-mercier@chu-montpellier.fr; 9 Laboratory of Biochemistry, Montpellier University Hospital, CEDEX 5, 34090 Montpellier, France

**Keywords:** heart failure, multivariable predictive score model, predicting mortality, rehospitalisations

## Abstract

**Background**: Acute heart failure (AHF) represents a leading cause of unscheduled hospital stays, frequent rehospitalisations, and mortality worldwide. The aim of our study was to develop a bedside prognostic tool, a multivariable predictive risk score, that is useful in daily practice, thus providing an early prognostic evaluation at admission and an accurate risk stratification after discharge in patients with AHF. **Methods**: This study is a subanalysis of the STADE HF study, which is a single-centre, prospective, randomised controlled trial enrolling 123 patients admitted to hospital for AHF. Here, 117 patients were included in the analysis, due to data exhaustivity. Regression analysis was performed to determine predictive variables for one-year mortality and/or rehospitalisation after discharge. **Results**: During the first year after discharge, 23 patients died. After modellisation, the variables considered to be of prognostic relevance in terms of mortality were (1) non-ischaemic aetiology of HF, (2) elevated creatinine levels at admission, (3) moderate/severe mitral regurgitation, and (4) prior HF hospitalisation. We designed a linear model based on these four independent predictive variables, and it showed a good ability to score and predict patient mortality with an AUC of 0.84 (95%CI: 0.76–0.92), thus denoting a high discriminative ability. A risk score equation was developed. During the first year after discharge, we observed as well that 41 patients died or were rehospitalised; hence, while searching for a model that could predict worsening health conditions (i.e., death and/or rehospitalisation), only two predictive variables were identified: non-ischaemic HF aetiology and previous HF hospitalisation (also included in the one-year mortality model). This second modellisation showed a more discrete discriminative ability with an AUC of 0.67 (95% C.I. 0.59–0.77). **Conclusions**: The proposed risk score and model, based on readily available predictive variables, are promising and useful tools to assess, respectively, the one-year mortality risk and the one-year mortality and/or rehospitalisations in patients hospitalised for AHF and to assist clinicians in the management of patients with HF aiming at improving their prognosis.

## 1. Introduction

Heart failure (HF) is a major public health challenge with an increasing incidence and prevalence worldwide and is the main cause of hospitalisation and subsequent death and rehospitalisation [1,2].

HF should not be considered as a single pathological diagnosis but as a clinical syndrome [3], mostly due to a structural and/or functional abnormality of the heart that results in elevated intracardiac pressures and/or inadequate cardiac output at rest and/or during exercise [4].

HF severity can be assessed using the New York Heart Association (NYHA) classification and the American College of Cardiology Foundation/American Heart Association (ACCF/AHA) classification [4].

The former describes the symptomatic status of the disease and the exercise capacity of patients on a scale from I to IV: Class I: no limitation of physical activity; Class II: slight limitation of physical activity; Class III: marked limitation of physical activity; and Class IV: symptoms occur even at rest, discomfort with any physical activity [1].

The latter analyses and categorises the development and progression of the disease according to the presence of HF symptoms and signs and cardiac structural changes into four stages: Stage A, patients at high risk for HF but without structural heart disease or symptoms of HF; Stage B, structural heart disease but without signs or symptoms of HF; Stage C, structural heart disease with prior or current symptoms of HF; and Stage D, refractory HF requiring specialised interventions [4,5].

Heart failure is usually divided into chronic heart failure (CHF) and acute heart failure (AHF). CHF describes those who have already had an established diagnosis of HF or those with a more gradual onset of symptoms. If CHF deteriorates, either suddenly or slowly, the episode may be described as decompensated HF [5]. This can result in hospital admission or treatment with intravenous diuretic therapy [5]. AHF refers to an abrupt onset of symptoms and clinical signs of heart failure leading to an unplanned hospital admission and stay. Patients with AHF should be evaluated immediately and treated as early as possible because of a poor prognosis [5]. AHF is described either as an acute decompensation of a previous heart failure, the so-called acute decompensated heart failure (ADHF), or as de novo AHF that refers to first episode of AHF or the abrupt onset of symptoms in patients with no prior history of cardiac dysfunction [3].

From a pathophysiological point of view, there are pleiotropic precipitating factors that can contribute to both ADHF and de novo AHF onset.

These factors are multifactorial and can be broadly categorised into cardiac and non-cardiac causes. The former include conditions such as coronary heart disease (CHD), hypertension, valvular heart disease, and arrhythmias; the latter comprise non-cardiac conditions (including chronic kidney disease, chronic obstructive pulmonary disease, diabetes, anaemia, and infections) and also surgical interventions and perioperative complications or lifestyle modifications (treatment non-compliance or dietary non-adherence) [6,7].

These cardiac and non-cardiac conditions are usually comorbidities in patients with AHF and play a pivotal role by triggering either de novo AHF or perpetuating and contributing to the exacerbation of decompensation episodes (ADHF).

HF is classified into three categories based on the left ventricular ejection fraction (EF) range: HF with reduced (HFrEF) ≤ 40%, mildly reduced (HFmrEF) 41–49%, and preserved EF (HFpEF) ≥ 50% [1,3].

AHF also represents an issue for clinicians in terms of health service management, besides being a heavy socio-economic burden [8,9]. 

After hospitalisation, the discharge is estimated to be a “vulnerable period” for patients transferring from hospital to home, with poor outcomes characterised by high rates of readmission and/or death [10,11,12].

The natural history of HF comprises acute decompensation episodes and associated rehospitalisations with established or putative precipitating factors, which must be delineated, in order to assess the risk adequately and to improve management before and after the discharge of patients with AHF [13].

Different risk models for HF have been developed worldwide but are scarcely used because many data, considered mandatory, are sometimes not easy to obtain in clinical practice [14,15,16,17,18,19].

A reliable prognostic score, useful in daily clinical practice, would include variables that can be easily assessed at any time during hospitalisation from the emergency department to the ward.

In the present study, we propose to develop and evaluate a prognosis score based on variables that are routinely available, such as medical history (previous hospitalisation, aetiology of HF, biological markers), and a basic echocardiographic assessment [15].

These routine parameters can be rapidly and easily collected in daily clinical practice, thus resulting in concrete support for clinicians in the decision-making process and adequate allocation of resources.

## 2. Material and Methods

The present study was a post hoc subanalysis of the STADE HF study, a blinded prospective randomised controlled trial conducted in the Cardiological Department of a university hospital centre in Montpellier, France (NCT02963272) [20].

The present study obtained Institutional Review Board approval (IRB-MTP_2023_04_202301376) from the IRB committee of Montpellier University Hospital. All the patients signed an informed consent form and were free to abandon this study at any time. 

**Figure 1:**
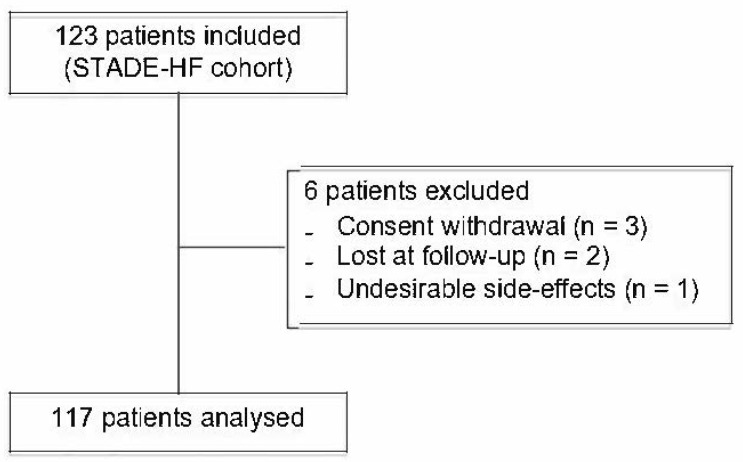
Study flowchart showing selection of patients included in the analyses.

Briefly, all the patients admitted for AHF with a preserved or altered left ventricular ejection fraction (LVEF) between January 2017 and August 2018 were enrolled in this study. The exclusion criteria were as follows: participation in another study, pregnancy or breastfeeding, or refusal. In total, 123 patients were included. Six patients were excluded due to consent withdrawal or because of missing information/data (Figure 1).

The data were collected by analysing medical files and by phone with the cardiologist or general practitioner, the patient, or the family. 

These data, including baseline clinical characteristics, medical history, biomarkers, and ultrasound parameters, were identified and selected as variables to develop a multivariable predictive score. The baseline characteristics of the included population are summarised in Table 1.

These variables, known to be associated with an increased mortality and AHF rehospitalisations according to evidence-based medicine and the literature, were chosen to produce a predictive risk score that is accessible in daily clinical practice.

The primary endpoint of our study was patient mortality within one year after discharge following the index hospitalisation for AHF. The secondary endpoint was the occurrence of death and/or rehospitalisation during the year following hospital discharge. 

### Statistical Analysis

The baseline characteristics were described using the mean with the standard deviation and the median with the interquartile range (Q1–Q3) for quantitative variables and numbers and percentages for categorical ones. The groups were compared with a Wilcoxon–Mann–Whitney test for continuous variables and a chi square or Fisher exact test otherwise.

Univariate logistic regression was performed to determine the baseline characteristics predicting death one year after discharge. A multivariate model using backward selection on variables with a *p*-value < 0.20 in univariate analysis was implemented. The odds ratio (OR) with 95% confidence interval (95% CI) was reported, and the prediction score was based on regression coefficients. A ROC (receiver operating characteristic) curve was drawn by plotting the sensitivity against the specificity of the score results. The area under the ROC curve (AUC) was calculated along with its 95%CI. The threshold was determined with the Youden index to optimise both the sensitivity and the specificity. The statistical measures of the threshold performance (sensitivity, specificity) were calculated. We defined a grey zone for cutoffs with a sensitivity lower than 90% and a specificity lower than 90%. A two-curve (sensitivity, specificity) representation was provided for illustration. The grey zone was defined as the values that did not allow a 10% diagnostic tolerance. No validation set was used due to the sample size. 

The same analysis was conducted to predict death or rehospitalisation at one year.

The statistical significance threshold was set at 5%. Statistical analysis was performed using SAS software 9.04 (SAS Institute, Cary, NC, USA).

## 3. Results 

### 3.1. Study Population and Baseline Characteristics

Of the initial 123 patients, 6 were excluded and 117 patients were analysed (Figure 1). The baseline characteristics of the included population are summarised in Table 1. 

Within one year after discharge, 23 patients (19.7%) died (Table 1). Certain variables of the baseline characteristics measured at inclusion had a statistically significant difference between dead and alive patients. The deceased patients were significantly older (*p* < 0.049). 

Considering the biological markers, the median values for soluble suppression of tumourigenicity 2 (ST2) and N-terminal pro-B-type natriuretic peptide (NT-proBNB) were around two-fold higher in the mortality group compared to those in the alive group (*p* < 0.006 and *p* < 0.004, respectively). The median levels of creatinine were slightly yet significantly higher in the mortality group (*p* < 0.013). Out of 23 deceased patients, 82.61% did not present an ischaemic aetiology of HF, compared to 59.57% for alive patients (*p* < 0.0390). The glomerular filtration rate (GFR) was lower in the deceased group compared to that in the alive group (*p* < 0.025).

Out of 23 deceased patients, 69.57% showed moderate–severe mitral regurgitation compared to 40.45% in alive patients (*p* < 0.0126). Similarly, for previous HF hospitalisation, which was more frequent in the mortality group compared to the alive group, out of 23 deceased patients, 14 were previously hospitalised compared to less than half of the alive patients (36 patients out of 94) (*p* < 0.0498).

### 3.2. One-Year Mortality Analysis

A univariate regression analysis allowed the selection of 10 variables to consider in the multivariate statistical model (Supplementary Table S1). 

**Figure 2:**
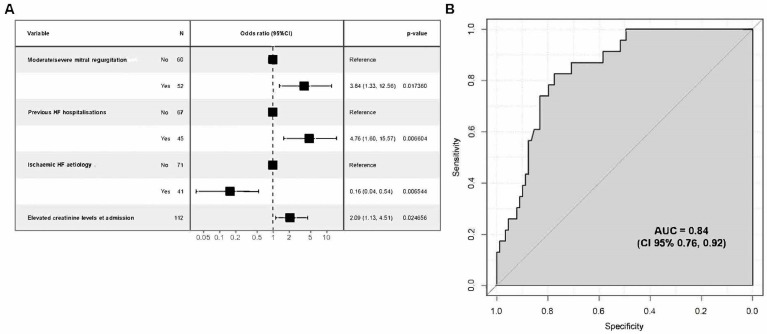
Regression analysis for risk assessment model on one-year mortality. (**A**) Odds ratio (CI 95%) for four statistically significant variables; (**B**) model performance evaluation ROC curve and AUC.

After modellisation, the variables considered to be of prognostic relevance were (i) non-ischaemic aetiology of HF, (ii) elevated creatinine levels at admission, (iii) moderate/severe grade of mitral regurgitation, and (iv) prior HF hospitalisation (Figure 2A). Hence, we designed a model that included these four independent predictive variables, and it showed a good predictive score with an AUC of 0.84 (95%CI: 0.77, 0.92), thus denoting a high discriminative ability (Figure 2B). 

Based on this model, a risk score equation was developed: [Score = −3.3138 − 1.8371 × non-ischaemic aetiology + 0.00738 × creatinine _µmol/L_ + 1.3460 × moderate/severe MR + 1.5598 × previous HF hospitalisation]. The best threshold for an ROC curve was −1.18 for this score with a sensitivity of 83% and a specificity of 78%. Values below this threshold are associated with a decreased mortality risk; inversely, values above −1.18 describe an increased mortality risk within 12 months after discharge.

**Figure 3:**
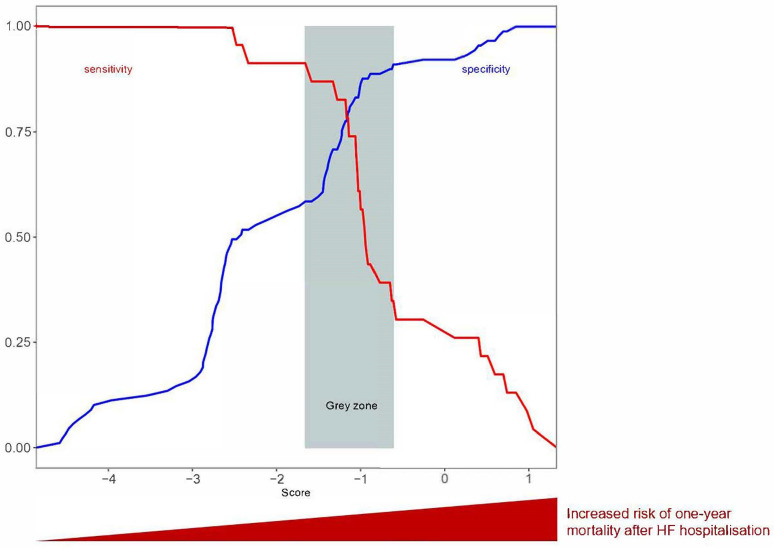
Grey zone analysis for sensitivity or specificity < 0.9.

An additional grey zone analysis on the threshold was performed (Figure 3). This approach determines a range of values for which no conclusion may be drawn. We defined inconclusive responses for score values with sensitivity <90% or specificity <90% (i.e., diagnostic tolerance of 10%). The grey zone for the proposed threshold ranged from −1.66 to −0.61 (Figure 3).

### 3.3. One-Year Mortality and/or Rehospitalisation Combined Event Risk Score and Validation 

The secondary endpoint was death and/or rehospitalisation during the first year after discharge. In our population, one of these two events occurred in 41 patients (Table 2). 

Baseline characteristics such as the N-terminus pro-brain natriuretic peptide (NT-proBNP) and having a previous HF hospitalisation had a statistically significant difference between dead/rehospitalised and alive/not hospitalised patients, respectively (*p* = 0.042). Out of 41 dead/rehospitalised patients, 58.54% had a previous HF hospitalisation compared to 34.21% for alive patients (*p* = 0.011).

**Figure 4:**
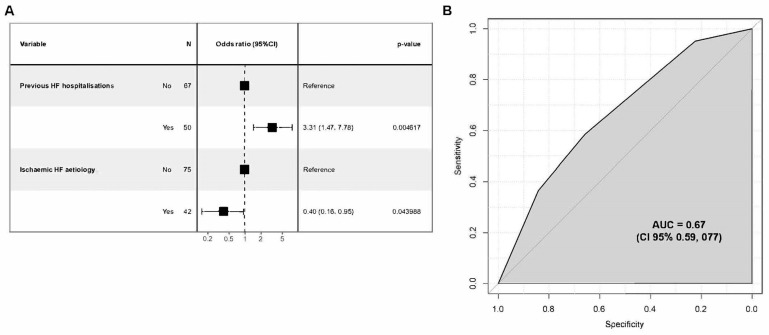
Regression analysis for risk assessment model on one-year mortality and/or rehospitalisation. (**A**) Odds ratio (CI 95%) for two statistically significant variables; (**B**) model performance evaluation ROC curve and AUC.

Again, 14 variables were initially considered, 8 of which were considered for the multivariate model (Supplementary Table S2). Of these, two variables turned out to be predictive of death or rehospitalisation in the 12 months following discharge: (i) non-ischaemic HF aetiology and (ii) prior HF hospitalisation (Figure 4A). In this secondary analysis, the results showed an AUC of 0.67 (95% C.I. 0.57, 0.76), thus indicating a discrete discriminative ability (Figure 4B). 

## 4. Study Limitations 

Firstly, this was a monocentric study.

Secondly, this study included a small sample size, which could lead to selection bias; therefore, it should be evaluated and validated with a larger population sample. It could be characterised from a statistical point of view to be “overfitting”, mainly because multiple candidate variables were used in a relatively small sample. 

Thirdly, there was a lack of external validation.

Lastly, a possible limitation could be that our patients were not admitted in the emergency department at first medical encounter but rather in the cardiologic intensive care unit, id est, a possible slight delay in some clinical and lab tests and a consequent delay in risk assessment. 

## 5. Discussion 

“Time is muscle”, as stated by Eugene Braunwald, highlights the importance of acting as soon as possible because the heart function deteriorates suddenly, especially if we think of the urgent clinical scenario in AHF and how time plays a role throughout the entire patient’s journey from hospital stay up to discharge and follow-up. 

Therefore, it is fundamental to have a fast bedside prognostic stratification of patients with AHF in an acute setting, considering the time needed by clinicians to treat and chiefly the patients’ care, procedures, and therapeutical interventions. Here, we propose a simple, easy-to-obtain evaluation of the risk of all-cause mortality at one year in patients admitted for acute HF. 

This result is derived from a one-year observational follow-up study on patients with AHF who were discharged after hospitalisation. Considering the many known risk factors for death and rehospitalisation in patients with AHF after hospital discharge [13], the main goal of our study was to identify routinely available variables that could best predict death and/or rehospitalisation for AHF during the year following discharge. This approach should help to tailor the best process for outpatient follow-up, leading to decisions determining subsequent treatment choices, specifically, the appropriate care setting (outpatient care, ward, or intensive care), therapeutical management (guideline-directed medical therapy and treatment optimisation, telemonitoring, frequent visits), or the possibility of seeing a specialised HF team (including dedicated nurses, pharmacists, or other healthcare providers).

Risk assessment is essential to better discriminate between high-risk and low-risk patients to avoid both overtreatment of low-risk patients and early, inadequate discharge of high-risk patients. High-risk patients need close, intensive, in-hospital and post-discharge monitoring, e.g., being transferred or hospitalised in an intensive care unit with continuous monitoring, with the involvement of dedicated networks and adequate resources. However, such patients do not usually receive the recommended medical therapy, due to comorbidities [21]. On the other hand, to avoid inadequate resources, low-risk patients could be potentially monitored in an ambulatory follow-up and require less surveillance compared to high-risk patients, and they may be discharged early [22].

To address the risk of death in the 12-month period after discharge, we found four variable modalities with a high predictive value, namely, (1) prior hospitalisation, (2) non-ischaemic aetiology of HF, (3) elevated creatinine level, and (4) presence of moderate/severe mitral regurgitation.

NT-proBNP and soluble suppression of tumourigenicity 2 (sST2) are biological markers universally acknowledged to be relevant to the diagnosis and prognosis of HF [23,24]. Although, as expected, elevated levels were observed in our deceased patient population, both at baseline and follow-up analyses, surprisingly, they did not appear to be of statistical significance in determining a prognostic score. We cannot exclude a lack of power. The only biomarker included in this score was the creatinine level, which has been well established as crucial for decades and could represent an integrative biomarker for both the cardiovascular system and comorbidities, reducing the additional value of other biomarkers.

Unfortunately, some potentially meaningful variables for the risk assessment of death and/or rehospitalisation were not included due to missing data, namely, BMI, glycaemia, HbA1c, ferritin, transferrin saturation, elevated blood urea nitrogen, and total bilirubin level. These are well known to be prevalent and frequent comorbidities in HF and, especially, independent predictors of rehospitalisation and mortality, thus meaning and leading to a worse outcome [1,8,9,10,11,13,15,17].

Even though we did not use the latter variables, our composite score demonstrated a high discriminative power to predict mortality within the year following discharge in patients with AHF, hence proving to be a reliable score. This score appears all the more usable in daily practice as only four universal variables, easy to obtain quickly, are mandatory.

Previous hospitalisation had an important prognostic role, as demonstrated by many other studies, being a strong predictor of mortality and repeated rehospitalisations [25,26,27].

The non-ischaemic aetiology correlated with a higher risk of rehospitalisation and death, as observed in a comparative analysis between patients with AHF of ischaemic and non-ischaemic aetiology from the “OP-AHF” registry, a single-centre study enrolling 122 patients; similarly, the same was also observed in a subanalysis of the Spanish Network for the Study of Heart Failure II registry (REDINSCOR II), a multicentre study with a larger population sample (1830 patients with AHF) [28,29]. An elevated creatinine level and prior HF hospitalisation were associated with a poor outcome as observed by Ruigómez et al. [30].

Increased creatinine serum levels reflect a decline in kidney function, the so-called WRF (worsening renal function). WRF is known to be associated with cardiovascular complications in the short term and the long term, thus leading to both rehospitalisation and mortality. Importantly, it has been observed as the main prognostic variable in many scores such as ADHERE [31], OPTIMIZE HF [32], and APACHE-HF [33].

The mechanisms that may cause WRF in patients with HF are multiple and are incompletely understood, possibly being neurohormonal activation, decreased renal perfusion, and intrarenal mechanisms involving increased endothelin and/or adenosine release. Medical treatment may also have a cause-and-effect relationship; for instance, daily intravenous furosemide dose, history of chronic kidney disease, NYHA class, and LVEF can be considered independent predictors of WRF [34].

Two out of the four variables in our score, a high creatinine level and a prior hospitalisation, were independently associated with a higher one-year mortality rate in the ESC-HF pilot study [35], a multicentre European survey with a large population sample.

This can partly overcome some of our study’s limitations, in particular, small population sample and monocentre study.

Another important aspect shown in the European survey is the higher rehospitalisation rate at one year in patients with acute heart failure compared to those with chronic heart failure, thus demonstrating the real value of a score that can estimate the rehospitalisation risk at one year in patients with AHF. 

In the FINN AVKA study [12], a multicentre study with 620 patients hospitalised for AHF, one of the independent predictors of 1-year mortality was renal dysfunction. In that study, the patients were divided into ADHF and de novo groups: the one-year mortality was higher in the ADHF group, and 40% of the ADHF group had a previous hospitalisation. In the ADHF group, the population was older and had more comorbidities (chronic kidney disease, valvular disease, diabetes), meaning that the population was frail and more prone to repeated hospitalisations. This result is in line with ours; previous hospitalisation correlates with a higher risk of mortality. The de novo patients had coronary acute syndrome as a precipitating factor unlike the ADHF group, meaning that, as in our study, episodes of decompensations were mainly explained by a non-ischaemic aetiology, thus being a negative prognostic factor of possible future rehospitalisations or a higher mortality risk.

Renal dysfunction is an independent variable related to one-year mortality even in the EFICA study [11]. The EFICA study, a multicentre French study, performed in intensive care units or intensive cardiologic care units, investigated short-term and long term-mortality, 4-week and one-year mortality, respectively, and subdivided patients into a cardiogenic shock group and a non-cardiogenic shock group. In that study, at baseline, an equal percentage of participants showed a history of either ischaemic heart disease or non-ischaemic heart disease, but, conversely to our study, mortality was higher in patients with an ischaemic aetiology. An ischaemic aetiology was observed in both patients with cardiogenic shock and patients with no cardiogenic shock, meaning that cardiogenic shock cannot be considered only as a complication of ischaemic heart disease. Thus, non-ischaemic aetiology plays a role, as observed in our study, and moreover, the number of patients presenting with cardiogenic shock of non-ischaemic aetiology was slightly higher than that of those with an ischaemic aetiology, 46 vs. 42.

In a more robust study, the Acute Decompensated Heart Failure National Registry (ADHERE) [14], the in-hospital mortality was the main aim. Among 80 variables collected in the ADHERE registry, 39 were selected and then analysed; surprisingly, only three of them proved to be prognostically significant in terms of mortality: high admission levels of blood urea nitrogen, low admission systolic blood pressure, and high admission levels of serum creatinine. Though the main aim of that study was in-hospital mortality and not one-year mortality, renal dysfunction was present. Hence, that study underlines how clinicians should pay attention to renal dysfunction both during hospitalisation, due to in-hospital mortality, and also after discharge. Contrary to our study, it used a large population sample and even more variables were selected, but, as in our study, renal dysfunction was the most relevant in terms of mortality.

Renal dysfunction results in further congestion and causes neurohormonal activation, which are factors associated with adverse outcomes in patients with heart failure.

Cardiorenal syndrome needs to be well recognised as soon as possible to optimise therapy in patients with HF in order to reduce associated hospitalisations and rehospitalisations, but mostly the related mortality [31].

Patients with AHF generally present signs and symptoms of systemic and pulmonary congestion and/or present a low cardiac output. Congestion is, hence, a problem of volume overload resulting from increased ventricular filling pressure frequently observed together with neurohormonal activation. Most hospitalisations and rehospitalisations are due more to clinical congestion than to low cardiac output [36,37].

Congestion has been always considered a challenge because, on one hand, diuretics could be prescribed, but on the other, renal function deters the prescription of diuretics. Signs of increased residual congestion, as evaluated by Pagnesi et al., even after diuretic use, could be evaluated through moderate/severe MR, and it has been associated as a negative prognostic factor [38]. Additionally, FMR (functional mitral regurgitation), regardless of aetiology, either ischaemic or not, has been strongly associated with a high rate of rehospitalisation and mortality [39,40,41]. Our results agree with these pathophysiological mechanisms, with the inclusion of the presence of moderate/severe MR in the predictive model for death within one year after discharge. 

A monocentric Italian study, the ACUTE HF score study [16], with a relatively small population sample had as its endpoint mortality at 30 days, 6 months, and 5 years. The results shown in that study were prognostically similar to those of our study, e.g., the presence of valvular heart disease including more-than-moderate mitral regurgitation, previous hospitalisation for HF, raised serum creatinine, and non-ischaemic AHF. The other variables independently correlated with mortality in their study were a history of stroke or TIA, low LVEF, and the use of non-invasive ventilation. It could be interesting to compare, as an external validation, our score in this ACUTE HF score study population. The fact that this variable is not included in our second proposed model hints that it has a major role in death instead of in rehospitalisations. 

Specifically, the risk assessment model for a death and/or rehospitalisation event within one year after discharge, included only two variables: non-ischaemic aetiology and previous HF hospitalisation. This model showed less predictive value than the one-year mortality-related model after discharge.

In our study, the model for one-year mortality and/or rehospitalisation showed a discrete discriminative ability (AUC 0.67). The same combined endpoint was present in a multicentre study with a larger population sample, the Coordinating Study Evaluating Outcomes of Advising and Counselling in Heart Failure (COACH) study [42]. Renal dysfunction was also highly predictive of the combined endpoint of HF hospitalisation or death. Other strong predictors for this endpoint were sex, myocardial infarction, serum sodium, and previous HF hospitalisation. The latter variable, considered as a strong predictor in the COACH study, is one of the two variables identified in our model.

The ability to calculate a risk score for patients with AHF greatly contributes to an improvement in HF clinical management. The score developed in this study showed favourable preliminary results, with a sensitivity of 83% and a specificity of 78% for a score threshold of −1.18. Nevertheless, future studies with a larger and external population are required to validate the proposed risk score.

The strength of the one-year mortality risk score is that it comprises only four variables: prior hospitalisation, non-ischaemic aetiology, elevated creatinine level, and moderate/severe mitral regurgitation. These parameters are easily available, fast to acquire, and not expensive even in an acute care setting. Ultimately, the risk score can contribute to the development of a daily routine tool for bedside evaluation and provide early management support for clinicians, complementing their medical judgement and thus leading to a risk-adjusted management of patients with HF.

## 6. Conclusions

The pathophysiology and mechanisms of AHF, especially inflammation and neurohormonal activation, are universal.

Our proposed model for evaluating one-year mortality risk relies on parameters that are routinely available, providing a new and promising tool in clinical practice. The strength of our model is that it is an easy, simple, bedside tool constituted of four variables, namely, (1) prior hospitalisation, (2) non-ischaemic HF aetiology, (3) elevated creatinine level, and (4) presence of moderate/severe mitral regurgitation that are used in every setting from the emergency department to the cardiology ward.

Further investigations are needed to compare our model using a larger sample, different populations, and a multicentric method and mostly to compare its efficacy with that of the already available scores.

In the future, this model can give rise to a reliable and useful tool to be implemented in daily clinical practice to improve care management and diagnostic and therapeutic pathways both during hospitalization and at discharge.

## Figures and Tables

**Table 1:** Baseline characteristics of general population and univariate analysis of variables between alive group and dead group.

Variable	*n*	Total Population	*n*	Deceased Patients	*n*	Alive Patients	*p*
**Age (years)**, median (Q1; Q3)	117	78.00 (66.00; 84.00)	23	83.00 (72.00; 87.00)	94	75.50 (65.00; 84.00)	0.0485
**Sex**, ***n*** (%)	117		23		94		0.2493
Female		48 (41.03)		7 (30.43)		41 (43.62)	
Male		69 (58.97)		16 (69.57)		53 (56.38)	
**BMI (kg/m^2^)**, median (Q1; Q3)	111	27.18 (23.59; 30.86)	22	27.51 (24.26; 29.96)	89	27.18 (23.43; 30.86)	0.6977
**Non-ischaemic aetiology of HF**, (%)	117	75 (64.10)	23	19 (82.61)	94	56 (59.57)	0.0390
**Soluble suppression of tumourigenicity 2 (sST2) (ng/mL)**, median (Q1; Q3)	117	95.50 (51.40; 222.00)	23	184.00 (112.00; 251.00)	94	80.65 (50.90; 194.00)	0.0055
**N-terminus pro-brain natriuretic peptide (NT-proBNP) (pg/mL)**, median (Q1; Q3)	117	4004.00 (2310; 7788)	23	7217.00 (4418; 21,694)	94	3739.00 (2099; 6945)	0.0036
**Serum creatinine (µmol/L)**, median (Q1; Q3)	117	108.00 (89.00; 141.00)	23	127.00 (108.00; 180.00)	94	105.50 (86.00; 135.00)	0.0132
**Haemoglobin (g/dL)**, median (Q1; Q3)	116	12.55 (11.00; 14.20)	23	12.00 (10.70; 13.10)	93	12.60 (11.00; 14.40)	0.1383
**LVEF** (**%**), ***n*** (%)	112	44.00 (30.00; 51.00)	23	40.00 (28.00; 52.00)	89	45.00 (30.00; 50.00)	0.7831
**Glomerular filtration rate (GFR) (mL/min/1.73 m^2^)**, median (Q1; Q3)	117	54.00 (38.00; 67.00)	23	43.00 (28.00; 57.00)	94	55.00 (41.00; 71.00)	0.0249
**Valvular heart disease (VHD)**, *n* (%)	112	86 (76.79)	23	20 (86.96)	89	66 (74.16)	0.1950
**Moderate–severe mitral regurgitation**, *n* (%)	112	52 (46.43)	23	16 (69.57)	89	36 (40.45)	0.0126
**Previous HF hospitalisation**, *n* (%)	117	50 (42.74)	23	14 (60.87)	94	36 (38.30)	0.0498

**Table 2:** Univariate analysis of variables between alive/non-rehospitalised group and deceased/rehospitalised groups.

Variable	*n*	Alive or No Rehospitalisation during First Year	*n*	Death or Rehospitalisation during First Year	*p*
**Age (years)**, median (Q1; Q3)	76	74.00 (65.50; 83.50)	41	80.00 (72.00; 87.00)	0.0624
**Sex**, ***n*** (**%**)	76		41		0.4733
Female		33 (43.42)		15 (36.59)	
Male		43 (56.58)		26 (63.41)	
**BMI (kg/m^2^)**, median (Q1; Q3)	73	27.26 (23.67; 31.02)	38	27.11 (23.59; 29.70)	0.6060
**Non-ischaemic aetiology of HF**	76	45 (59.21)	41	30 (73.17)	0.1331
**Soluble suppression of tumourigenicity 2 (sST2) (ng/mL)**, median (Q1; Q3)	76	88.10 (50.95; 203.50)	41	117.00 (57.00; 250.00)	0.1772
**(N-terminus pro-brain natriuretic peptide) NT-proBNP (pg/mL)**, median (Q1; Q3)	76	3585.00 (2100.00; 7050.00)	41	5733.00 (3032.00; 11,347.00)	0.0423
**Serum creatinine (µmol/L)**, median (Q1; Q3)	76	105.50 (89.00; 140.00)	41	113.00 (94.00; 147.00)	0.3930
**Haemoglobin (Hb) (g/dL)**, median (Q1; Q3)	76	12.60 (11.00; 14.65)	40	12.25 (11.05; 13.65)	0.3511
**Glomerular filtration rate (GFR) (mL/min/1.73 m^2^)**, median (Q1; Q3)	76	54.00 (39.50; 70.00)	41	47.00 (30.00; 65.00)	0.6093
**LVEF** (**%**) median (Q1; Q3)	71	43.00 (30.00; 50.00)	41	45.00 (32.00; 60.00)	0.5079
**Valvular heart disease**, *n* (%)	71	52 (73.24)	41	34 (82.93)	0.2421
**Moderate–severe mitral regurgitation**, *n* (%)	71	28 (39.44)	41	24 (58.54)	0.0509
**Previous HF hospitalisation**, *n* (%)	76	26 (34.21)	41	24 (58.54)	0.0112

## Data Availability

Data are available on reasonable request.

## Supplementary Materials

The following supporting information can be downloaded at: https://www.mdpi.com/article/10.3390/jcm13072018/s1, Table S1: Multivariate regression analysis for one-year mortality. Table S2: 6 variables model.
