# Supplementary material for: Predicting One-Year Mortality after Discharge Using Acute Heart Failure Score (AHFS)
Source: J Clin Med. 2024 Mar 30;13(7):2018. doi: 10.3390/jcm13072018 (PMC11012877; doi:10.3390/jcm13072018)
Supplement: Supplementary file 1 [file jcm-13-02018-s001.zip › jcm-2908863-supplementary.pdf]

Supplementary Data

Supplementary Table S1: Multivariate regression analysis for one-year mortality

variables	unit	n	OR_CI	pglobal
Non ischemic etiology		117	0.31 [0.10–0.98]	0.0469
Intermediate-severe mitral regurgitation		112	3.37 [1.26–9.00]	0.0156
Soluble suppression of tumorigenicity 2 (sST2)	100	117	2.20 [1.26–3.82]	0.0053
Age	5	117	1.23 [0.99–1.53]	0.0557
Previous Heart Failure hospitalisation		117	2.51 [0.98–6.38]	0.0541
N-terminal prohormone of brain natriuretic peptide (NT-proBNP)	1000	117	1.04 [1.00–1.07]	0.0515
Serum Creatinine	100	117	1.65 [0.93–2.94]	0.0884
Glomerular Filtration Rate (GFR)	10	117	0.81 [0.64–1.02]	0.0674
Serum total Bilirubin	10	101	1.31 [0.99–1.74]	0.0627
Hemoglobin (Hb)	10	116	0.22 [0.03–1.89]	0.1696

Supplementary Table S2: 6 variables model

Variables	unit	n	OR_IC	pglobal
**Soluble suppression of tumorigenicity 2 (sST2) (ng/mL)**	100.0	117	1.37 [0.88–2.13]	0.1700
**Serum Bilirubin**	10.0000	101	1.32 [0.98–1.77]	0.0648
**Age**	5.0000	117	1.15 [0.98–1.35]	0.0881
**Moderate-severe mitral regurgitation** *Yes* **vs.** *No*		112	2.17 [0.99–4.74]	0.0527
**Previous HF hospitalisation** *Yes* **vs.** *No*		117	2.71 [1.24–5.93]	0.0122
**Ischemic etiology Yes vs. No**		117	0.53 [0.23–1.22]	0.1359
